# *LINC00673* rs11655237 C>T confers neuroblastoma susceptibility in Chinese population

**DOI:** 10.1042/BSR20171667

**Published:** 2018-02-08

**Authors:** Zhuorong Zhang, Yitian Chang, Wei Jia, Jiao Zhang, Ruizhong Zhang, Jinhong Zhu, Tianyou Yang, Huimin Xia, Yan Zou, Jing He

**Affiliations:** 1 Department of Pediatric Surgery, Guangzhou Institute of Pediatrics, Guangzhou Women and Children’s Medical Center, Guangzhou Medical University, Guangzhou 510623, Guangdong, China; 2 College of Clinical Medicine, Jilin University, Changchun 130021, Jilin, China; 3 Department of Pediatric Surgery, The First Affiliated Hospital of Zhengzhou University, Zhengzhou 450052, Henan, China; 4 Molecular Epidemiology Laboratory and Department of Laboratory Medicine, Harbin Medical University Cancer Hospital, Harbin 150040, Heilongjiang, China

**Keywords:** LINC00673, neuroblastoma, polymorphism, susceptibility

## Abstract

Neuroblastoma, which accounts for approximately 10% of all pediatric cancer-related deaths, has become a therapeutic challenge and global burden attributed to poor outcomes and mortality rates of its high-risk form. Previous genome-wide association studies (GWASs) identified the *LINC00673* rs11655237 C>T polymorphism to be associated with the susceptibility of several malignant tumors. However, the association between this polymorphism and neuroblastoma susceptibility is not clear. We genotyped *LINC00673* rs11655237 C>T in 393 neuroblastoma patients in comparison with 812 age-, gender-, and ethnicity-matched healthy controls. We found a significant association between the *LINC00673* rs11655237 C>T polymorphism and neuroblastoma risk (TT compared with CC: adjusted odds ratio (OR) =1.80, 95% confidence interval (CI) =1.06–3.06, *P*=0.029; TT/CT compared with CC: adjusted OR =1.31, 95% CI =1.02–1.67, *P*=0.033; and T compared with C: adjusted OR =1.29, 95% CI =1.06–1.58, *P*=0.013). Furthermore, stratified analysis indicated that the rs11655237 T allele carriers were associated with increased neuroblastoma risk for patients with tumor originating from the adrenal gland (adjusted OR =1.51, 95% CI =1.06–2.14, *P*=0.021) and International Neuroblastoma Staging System (INSS) stage IV disease (adjusted OR =1.60, 95% CI =1.12–2.30, *P*=0.011). In conclusion, we verified that the *LINC00673* rs11655237 C>T polymorphism might be associated with neuroblastoma susceptibility. Prospective studies with a large sample size and different ethnicities are needed to validate our findings.

## Introduction

Neuroblastoma, originating from the developing sympathetic nervous system, is the most common extracranial tumor of infancy and childhood. The median age at diagnosis is approximately 18 months [1]. It is a fatal solid cancer accounting for approximately 8–10% of all childhood malignancies [1–3] and contributes to approximately 10–15% of all cancer-related deaths in children [4]. Despite intensive multimodal treatments [5], 5-year event-free survival for high-risk (approximately 50% of all cases) neuroblastoma patients remains less than 50% [6,7], and the 5-year survival rate for all neuroblastoma patients is approximately 50% [8]. Due to the rarity of neuroblastoma, strict associations are difficult to certify, and no particular environmental exposure has been involved in the development of this disease [1]. Thus, neuroblastoma remains a huge therapeutic challenge and global burden.

Previous genome-wide association studies (GWASs) of pancreatic ductal adenocarcinoma have identified a long intergenic non-coding RNA (lincRNA) *LINC00673* rs11655237 C>T (also reported as G>A elsewhere) polymorphism at 17q25.1 region to be significantly associated with the risk of pancreatic cancer [9]. It was the most significant polymorphism in the *LINC00673* [9]. Further studies have proven that the rs11655237 in exon 4 of *LINC00673* forms a target site for *miR-1231* binding, which weakens the tumor suppressing function of *LINC00673* in an allele-specific manner and thus endows pancreatic cancer with susceptibility [10]. Moreover, *LINC00673* has been identified to be associated with non-small-cell lung cancer (NSCLC) [11–13], gastric cancer [14], tongue squamous cell carcinoma [15], and breast cancer [16] consecutively. Together, these findings indicated that the *LINC00673* rs11655237 appears to be implicated in the development of broad-spectrum tumors and also involved in cell homeostasis maintenance. However, the association between neuroblastoma susceptibility and *LINC00673* rs11655237, is not yet reported.

To comprehensively evaluate and provide new insights into the impact of *LINC00673* rs11655237 C>T polymorphism on neuroblastoma susceptibility, we performed a hospital-based case–control study using data from a Chinese Han population composed of 393 neuroblastoma cases and 812 age-, gender-, ethnicity-matched cancer-free controls.

## Methods

### Study subjects

The subjects included were described in our previous studies [17–20]. A total of 393 histopathological cases of primary neuroblastoma and 812 cancer-free controls from Guangdong province (South China) and Henan province (North China) were recruited in the current study (Supplementary Table S1). Briefly, all the 393 cases were newly confirmed and histopathologically diagnosed as neuroblastoma patients without progressive disease and previous treatments before the collection of clinical classification. The cases were genetically unrelated Chinese Han children who received treatments at the Guangzhou Women and Children’s Medical Center between February 2010 and March 2017, and at the First Affiliated Hospital of Zhengzhou University between August 2011 and April 2017. Age-, gender-, and ethnicity-matched controls were randomly recruited from children undergoing routine medical examination at the same center during the same period. The parents or guardians of the children provided written informed consent for the children’s participation in the present study. The current study was approved by the Institutional Review Board of both centers and performed in accordance with the study protocol.

### Genotyping

DNA samples were extracted as previously described [21]. Briefly, DNA samples were diluted to a stock concentration of 10 ng/μl and added to 96-well plates. Genotyping for the *LINC00673* rs11655237 C>T polymorphism was carried out in a 384-well plate using TaqMan Real-Time PCR method as described elsewhere [22–24]. For quality control and accuracy of genotyping results, approximately 10% of the samples were randomly selected and re-genotyped. The results were 100% concordant.

### Statistical analysis

Comparison of the differences in the demographic and genotypic information between neuroblastoma cases and controls was performed by chi-square test. Hardy–Weinberg equilibrium for controls was analyzed by goodness-of-fit chi-square test. To estimate the strength of association between rs11655237 C>T polymorphism and neuroblastoma risk, unconditional univariate and multivariate logistic regression analyses were conducted and adjusted for age and gender, while odds ratios (ORs) and 95% confidence intervals (CIs) were used. Further stratified analysis was performed by age, gender, the International Neuroblastoma Staging System (INSS) clinical stages [25], and tumor sites. *P*-values <0.05 were considered as statistically significant. All statistical analyses were two-sided and were calculated by SAS software (version 9.1; SAS Institute, Cary, NC, U.S.A.).

## Results

### Demographic characteristics

The demographic characteristics of the included participants are summarized in Supplementary Table S1. No statistically significant differences were observed between neuroblastoma cases and controls regarding age (*P*=0.229, *P*=0.484, *P*=0.437) and gender (*P*=0.510, *P*=0.196, *P*=0.836) for Guangdong, Henan, and combined subjects, respectively. According to the INSS criteria, 69 (17.56%), 93 (23.66%), 68 (17.30%), 143 (36.39%), and 11 (2.80%) patients were diagnosed with clinical stages I, II, III, IV, and 4s disease, respectively. With respect to tumor sites, 153 (38.93%) neuroblastomas occurred in the adrenal glands, 87 (22.14%) in retroperitoneal regions, 109 (27.74%) in the mediastinum, and 36 (9.16%) in other regions.

### *LINC00673* rs11655237 C>T polymorphism and neuroblastoma susceptibility

The *LINC00673* rs11655237 genotype frequencies and their association with the susceptibility of neuroblastoma are presented in Table 1. A total of 391 of the included neuroblastoma cases and all controls (812) were successfully genotyped. We found that the rs11655237 T allele carriers (CT/TT compared with CC: adjusted OR = 1.31, 95% CI = 1.02–1.67, *P*=0.033; and T compared with C: adjusted OR = 1.29, 95% CI = 1.06–1.58, *P*=0.013), especially mutated-type homozygote carriers (TT compared with CC: adjusted OR = 1.80, 95% CI = 1.06–3.06, *P*=0.029), were significantly associated with an increased risk of neuroblastoma when compared with wild-type homozygote carriers.

**Table 1:** Genotype distributions of *LINC00673* rs11655237 C>T polymorphism and neuroblastoma susceptibility

Genotype	Cases (*n*=391)	Controls (*n*=812)	*P*^1^	Crude OR (95% CI)	*P*	Adjusted OR (95% CI)^2^	*P*^2^
rs11655237 C>T (HWE = 0.831)							
CC	218 (55.75)	505 (62.19)		1.00		1.00	
CT	146 (37.34)	272 (33.50)		1.24 (0.96–1.61)	0.096	1.24 (0.96–1.60)	0.099
TT	27 (6.91)	35 (4.31)		**1.79 (1.06–3.03)**	**0.031**	**1.80 (1.06–3.06)**	**0.029**
Additive			0.041	**1.29 (1.05–1.57)**	**0.014**	**1.29 (1.06–1.58)**	**0.013**
Dominant	173 (44.25)	307 (37.81)	0.033	**1.31 (1.02–1.67)**	**0.033**	**1.31 (1.02–1.67)**	**0.033**
Recessive	364 (93.09)	777 (95.69)	0.057	1.65 (0.98–2.76)	0.059	1.66 (0.99–2.79)	0.054
C	582 (74.42)	1282 (78.94)		1.00		1.00	
T	200 (25.58)	342 (21.06)	0.013	**1.29 (1.06–1.57)**	**0.013**	**1.29 (1.06–1.58)**	**0.013**

1 *χ*^2^ test for genotype distributions between neuroblastoma cases and cancer-free controls.

2 Adjusted for age and gender. Results shown in bold if 95% CI excluded 1 or *P*<0.05.

### Stratified analysis

Next, the included participants were stratified by age, gender, tumor sites, and INSS stages. We further estimated the effects of the variant genotype of the rs11655237 C>T polymorphism on the neuroblastoma risk amongst the different strata (Table 2). We found that the rs11655237 CT/TT genotype carriers were significantly associated with an increased risk of a tumor originating in the adrenal gland (adjusted OR = 1.51, 95% CI = 1.06–2.14, *P*=0.021). Furthermore, we also found that the carriers of the CT/TT genotypes had a significantly increased risk of INSS clinical stage IV disease (adjusted OR = 1.60, 95% CI = 1.12–2.30, *P*=0.011) when compared with the carriers of the CC genotype.

**Table 2:** Stratification analysis for the association between *LINC00673* rs11655237 C>T polymorphism and neuroblastoma susceptibility for combined subjects

Variables	rs11655237 (cases/controls)		Crude OR	*P*	Adjusted OR^1^	*P*^1^
	CC	CT/TT	(95% CI)		(95% CI)	
Age, months						
≤18	72/194	53/111	1.29 (0.84–1.97)	0.245	1.29 (0.84–1.97)	0.246
>18	146/311	120/196	1.30 (0.97–1.76)	0.083	1.30 (0.96–1.76)	0.085
Gender						
Female	89/210	79/132	1.41 (0.97–2.05)	0.070	1.41 (0.97–2.05)	0.071
Male	129/295	94/175	1.23 (0.89–1.70)	0.215	1.23 (0.89–1.70)	0.216
Site of origin						
Adrenal gland	80/505	73/307	**1.50 (1.06–2.13)**	**0.022**	**1.51 (1.06–2.14)**	**0.021**
Retroperitoneal	49/505	36/307	1.21 (0.77–1.90)	0.413	1.21 (0.77–1.91)	0.402
Mediastinum	65/505	44/307	1.11 (0.74–1.68)	0.606	1.11 (0.74–1.67)	0.618
Others	22/505	14/307	1.05 (0.53–2.08)	0.896	1.04 (0.53–2.07)	0.904
Clinical stages						
I + II + 4s	93/505	69/307	1.22 (0.87–1.72)	0.254	1.21 (0.86–1.70)	0.278
III + IV	115/505	95/307	1.36 (1.00–1.85)	0.050	1.35 (1.00–1.84)	0.054
III	43/505	25/307	0.96 (0.57–1.60)	0.865	0.95 (0.57–1.59)	0.854
IV	72/505	70/307	**1.60 (1.12–2.29)**	**0.010**	**1.60 (1.12–2.30)**	**0.011**

1 Adjusted for age and gender. Results shown in bold if 95% CI excluded 1 or *P*<0.05.

## Discussion

In this hospital-based case–control study, we evaluated the association of the GWAS-identified *LINC00673* rs11655237 C>T polymorphism with neuroblastoma susceptibility in 393 patients and 812 cancer-free controls. Our results revealed that the rs11655237 T allele significantly increased the risk of neuroblastoma. In addition, stratified analysis showed that the rs11655237 C>T polymorphism increased the risk of adrenal gland and clinical stage IV neuroblastoma. The results from the present study suggested that the rs11655237 T allele was positively associated with neuroblastoma and could confer neuroblastoma susceptibility.

Described as the largest subclass in the non-coding transcriptome in humans, lincRNAs are non-coding transcripts longer than 200 nts. In the past decade, the roles of lincRNAs in human disorders, including tumors, have attracted a lot of attention [26,27]. lincRNAs are known to play vital regulatory roles in various processes [28], including but not limited to imprinting (*H19* [29] and *KCNQ1OT1* [30]), metastasis (*MALAT1* [31], *HOTAIR* [32], and *COLDAIR* [33]), X inactivation (*XIST* [34]), deregulation of the tumor suppressors [35], and pseudogene pairing (*PTENP1* [36]). Therefore, aberrant expression and polymorphisms within lincRNAs have been associated with susceptibility to a range of human diseases, including cancers. Cancer-associated lincRNAs, which demonstrate developmental and tissue-specific expression properties along with aberrant regulation in various malignancies, may indicate new approaches for the diagnosis and treatment of cancer. Systematic identification of the expression patterns and characterization of lincRNAs together with their associated proteins might contribute to the development of lincRNA-targetted therapies for tumors and various human diseases.

*LINC00673* is located at 17q24.3, which is a chromosome region recently documented to have a high frequency of loss of heterozygosity [37], and is associated with pancreatic cancer susceptibility in individuals of European ancestry [9]. Further studies have indicated that the *LINC00673* rs11655237 variant, a germline C>T transition, can lead to a decrease in the level of *LINC00673* in cells, which may trigger SRC-ERK oncogenic signaling, while attenuating STAT1-dependent anti-oncogenic signaling. Consistent with these functional discoveries, researchers have observed significantly decreased pancreatic ductal adenocarcinoma susceptibility in the rs11655237 C allele carriers when compared with the rs116552337 T allele carriers [10,38]. Intriguingly, *LINC00673* has been proven to be associated with susceptibility, progression, and outcome of other malignancies as either a tumor suppressor or promoter. Shi et al. [39] found that up-regulation of *LINC00673* can promote tumor proliferation through LSD1 interaction and repression of Neurocalcin δ in NSCLC, which implied *LINC00673* as an oncogene in NSCLC. For the first time, Ma et al. [40] showed that *LINC00673* promoted NSCLC metastasis by binding with EZH2 that resulted in epigenetic silencing of *HOXA5*. The latter is a tumor suppressor gene that inhibits NSCLC cell metastasis via regulating cytoskeletal remodeling. Lu et al. [11] found a similar situation in which *LINC00673* regulated NSCLC proliferation, invasion, migration, and even epithelial–mesenchymal transition by sponging *miR-150-5p*. A study by Abdul-Rahman et al. [16] showed that *LINC00673* levels were modulated by hormone signaling and inversely associated with breast cancer survival. Huang et al. [14] reported that *LINC00673* was significantly up-regulated in gastric cancer. *LINC00673* overexpression induced cell proliferation and invasion and inhibited cell apoptosis. Research conducted by Yu et al. [15] showed that *LINC00673* was highly expressed in a significant proportion of human tongue squamous cell carcinoma samples and associated with poor prognosis.

Taken together, these findings shed light on an important functional interaction between *LINC00673* and malignancies. Therefore, it would be interesting to examine the role of *LINC00673* in neuroblastoma. With such purpose, we conducted the current study in the Chinese Han population. The current study has several important strengths. To the best of our knowledge, this is the first investigation to validate the association of neuroblastoma susceptibility with GWAS-identified polymorphism within *LINC00673*. The identification of *LINC00673* as an antitumor factor in neuroblastoma was based on discoveries in GWASs in several tumors. Although our findings showed that the association between rs11655237 and neuroblastoma susceptibility were obtained in Chinese Han populations and are consistent with previous reports in other malignancies, further studies on this and other tumors in a different ethnic group would be beneficial. In addition, it has been suggested that rs11655237 might have a potential role in the regulation of gene expression via an enhancer, promoter, or silencer mechanism [9]. Therefore, further exploration of this issue should be addressed. Additionally, it would be intriguing to validate the effects of rs11655237 variation on the development of neuroblastoma using *in vivo* experimental settings.

Several limitations should be addressed in the present study. First, although it was the first study on the association between *LINC00673* and neuroblastoma, we included only 393 neuroblastoma cases and 812 cancer-free controls. The relative small sample size may limit the statistical power. Replication studies from other centers with a larger sample size are encouraged to confirm the association. Second, only one polymorphism of the newly identified *LINC00673* was investigated in the current study. More polymorphisms, especially the potentially functional polymorphisms not yet contained in previous GWASs, remain to be explored. Third, due to the design of the retrospective study, information bias and selection bias might be unavoidable. Because of the lack of information on dietary intake, living environmental factors, and parental exposures, only frequency matching of neuroblastoma cases and controls by age and gender could be used to reduce these biases. Fourth, *in vitro* and *in vivo* experiments should be performed to interrogate mechanism(s) underlying the association in the future. Finally, although recruited from two centers, including residents in Southern and Northern China, participants were all Han Chinese, so the results should be cautiously extrapolated to other ethnic groups.

In conclusion, our results verified that the *LINC00673* rs11655237 C>T polymorphism was significantly associated with neuroblastoma susceptibility in the Chinese Han population, especially for children with neuroblastoma of the adrenal gland region and clinical stage IV. In the future, well-designed prospective studies with larger sample size including different ethnic populations, detailed information (including living environment, dietary intake, and parental exposures) and functional studies should be performed to strengthen our findings.

### Supporting information

**Supplemental Table 1:** Frequency distribution of selected characteristics in neuroblastoma cases and cancer-free controls

## Abbreviations

CI: confidence interval
GWAS: genome-wide association study
INSS: International Neuroblastoma Staging System
lincRNA: long intergenic non-coding RNA
NSCLC: non-small-cell lung cancer
OR: odds ratio

## References

[1] Shohet J. and Foster J. (2017) Neuroblastoma. BMJ 357, j1863 10.1136/bmj.j1863 28468760

[2] Brodeur G.M. and Bagatell R. (2014) Mechanisms of neuroblastoma regression. Nat. Rev. Clin. Oncol. 11, 704–713 10.1038/nrclinonc.2014.168 25331179 PMC4244231

[3] Siegel R.L., Miller K.D. and Jemal A. (2017) Cancer statistics, 2017. CA Cancer J. Clin. 67, 7–30 10.3322/caac.21387 28055103

[4] Maris J.M., Mosse Y.P., Bradfield J.P., Hou C., Monni S. (2008) Chromosome 6p22 locus associated with clinically aggressive neuroblastoma. N. Engl. J. Med. 358, 2585–2593 10.1056/NEJMoa0708698 18463370 PMC2742373

[5] Cordeau M., Belounis A., Lelaidier M., Cordeiro P., Sartelet H. (2016) Efficient killing of high risk neuroblastoma using natural killer cells activated by plasmacytoid dendritic cells. PLoS ONE 11, e0164401 10.1371/journal.pone.0164401 27716850 PMC5055353

[6] Yu A.L., Gilman A.L., Ozkaynak M.F., London W.B., Kreissman S.G. (2010) Anti-GD2 antibody with GM-CSF, interleukin-2, and isotretinoin for neuroblastoma. N. Engl. J. Med. 363, 1324–1334 10.1056/NEJMoa0911123 20879881 PMC3086629

[7] Yalcin B., Kremer L.C. and van Dalen E.C. (2015) High-dose chemotherapy and autologous haematopoietic stem cell rescue for children with high-risk neuroblastoma. Cochrane Database Syst. Rev. CD006301, 10.1002/14651858.CD006301.pub3 26436598 PMC8783746

[8] Peinemann F., van Dalen E.C., Tushabe D.A. and Berthold F. (2015) Retinoic acid post consolidation therapy for high-risk neuroblastoma patients treated with autologous hematopoietic stem cell transplantation. Cochrane Database Syst. Rev. 1, CD010685 25634649 10.1002/14651858.CD010685.pub2

[9] Childs E.J., Mocci E., Campa D., Bracci P.M., Gallinger S. (2015) Common variation at 2p13.3, 3q29, 7p13 and 17q25.1 associated with susceptibility to pancreatic cancer. Nat. Genet. 47, 911–916 10.1038/ng.3341 26098869 PMC4520746

[10] Zheng J., Huang X., Tan W., Yu D., Du Z. (2016) Pancreatic cancer risk variant in LINC00673 creates a miR-1231 binding site and interferes with PTPN11 degradation. Nat. Genet. 48, 747–757 10.1038/ng.3568 27213290

[11] Lu W., Zhang H., Niu Y., Wu Y., Sun W. (2017) Long non-coding RNA linc00673 regulated non-small cell lung cancer proliferation, migration, invasion and epithelial mesenchymal transition by sponging miR-150-5p. Mol. Cancer 16, 118 10.1186/s12943-017-0685-9 28697764 PMC5504775

[12] Tan Q., Yu Y., Li N., Jing W., Zhou H. (2017) Identification of long non-coding RNA 00312 and 00673 in human NSCLC tissues. Mol. Med. Rep. 16, 4721–4729 10.3892/mmr.2017.7196 28849087 PMC5647024

[13] Lu W., Zhang H., Niu Y., Wu Y., Sun W. (2017) Erratum to: Long non-coding RNA linc00673 regulated non-small cell lung cancer proliferation, migration, invasion and epithelial mesenchymal transition by sponging *miR-150-5p*. Mol. Cancer 16, 144 10.1186/s12943-017-0716-6 28851432 PMC5574224

[14] Huang M., Hou J., Wang Y., Xie M., Wei C. (2017) Long noncoding RNA LINC00673 is activated by SP1 and exerts oncogenic properties by interacting with LSD1 and EZH2 in gastric cancer. Mol. Ther. 25, 1014–1026 10.1016/j.ymthe.2017.01.017 28214253 PMC5383578

[15] Yu J., Liu Y., Gong Z., Zhang S., Guo C. (2017) Overexpression long non-coding RNA LINC00673 is associated with poor prognosis and promotes invasion and metastasis in tongue squamous cell carcinoma. Oncotarget 8, 16621–16632 28039470 10.18632/oncotarget.14200 PMC5369989

[16] Abdul-Rahman U., Gyorffy B. and Adams B.D. (2017) linc00673 (ERRLR01) is a prognostic indicator of overall survival in breast cancer. Transcription, 10.1080/21541264.2017.1329684 PMC5791815 28795861

[17] Zhang J., Lin H., Wang J., He J., Zhang D. (2017) LMO1 polymorphisms reduce neuroblastoma risk in Chinese children: a two-center case-control study. Oncotarget 8, 65620–65626 29029458 10.18632/oncotarget.20018 PMC5630358

[18] He J., Wang F., Zhu J., Zhang Z., Zou Y. (2017) The TP53 gene rs1042522 C>G polymorphism and neuroblastoma risk in Chinese children. Aging (Albany N.Y.) 9, 852–859 28275206 10.18632/aging.101196 PMC5391235

[19] He J., Zou Y., Wang T., Zhang R., Yang T. (2017) Genetic variations of GWAS-identified genes and neuroblastoma susceptibility: a replication study in Southern Chinese children. Transl. Oncol. 10, 936–941 10.1016/j.tranon.2017.09.008 29024823 PMC5704095

[20] He J., Wang F., Zhu J., Zhang R., Yang T. (2016) Association of potentially functional variants in the XPG gene with neuroblastoma risk in a Chinese population. J. Cell. Mol. Med. 20, 1481–1490 10.1111/jcmm.12836 27019310 PMC4956948

[21] He J., Zhang R., Zou Y., Zhu J., Yang T. (2016) Evaluation of GWAS-identified SNPs at 6p22 with neuroblastoma susceptibility in a Chinese population. Tumour Biol. 37, 1635–1639 10.1007/s13277-015-3936-7 26307394

[22] Li J., Zou L., Zhou Y., Li L., Zhu Y. (2017) A low-frequency variant in SMAD7 modulates TGF-beta signaling and confers risk for colorectal cancer in Chinese population. Mol. Carcinog. 56, 1798–1807 10.1002/mc.22637 28218435

[23] Lou J., Gong J., Ke J., Tian J., Zhang Y. (2017) A functional polymorphism located at transcription factor binding sites, rs6695837 near LAMC1 gene, confers risk of colorectal cancer in Chinese populations. Carcinogenesis 38, 177–183 28039327 10.1093/carcin/bgw204

[24] He J., Qiu L.X., Wang M.Y., Hua R.X., Zhang R.X. (2012) Polymorphisms in the XPG gene and risk of gastric cancer in Chinese populations. Hum. Genet. 131, 1235–1244 10.1007/s00439-012-1152-8 22371296

[25] Brodeur G.M., Pritchard J., Berthold F., Carlsen N.L., Castel V. (1994) Revisions of the international criteria for neuroblastoma diagnosis, staging and response to treatment. Prog. Clin. Biol. Res. 385, 363–369 7972232

[26] Tsai M.C., Spitale R.C. and Chang H.Y. (2011) Long intergenic noncoding RNAs: new links in cancer progression. Cancer Res. 71, 3–7 10.1158/0008-5472.CAN-10-2483 21199792 PMC3057914

[27] Gibb E.A., Brown C.J. and Lam W.L. (2011) The functional role of long non-coding RNA in human carcinomas. Mol. Cancer 10, 38 10.1186/1476-4598-10-38 21489289 PMC3098824

[28] Cabili M.N., Trapnell C., Goff L., Koziol M., Tazon-Vega B. (2011) Integrative annotation of human large intergenic noncoding RNAs reveals global properties and specific subclasses. Genes Dev. 25, 1915–1927 10.1101/gad.17446611 21890647 PMC3185964

[29] Leighton P.A., Ingram R.S., Eggenschwiler J., Efstratiadis A. and Tilghman S.M. (1995) Disruption of imprinting caused by deletion of the H19 gene region in mice. Nature 375, 34–39 10.1038/375034a0 7536897

[30] Pandey R.R., Mondal T., Mohammad F., Enroth S., Redrup L. (2008) Kcnq1ot1 antisense noncoding RNA mediates lineage-specific transcriptional silencing through chromatin-level regulation. Mol. Cell 32, 232–246 10.1016/j.molcel.2008.08.022 18951091

[31] Ji P., Diederichs S., Wang W., Boing S., Metzger R. (2003) MALAT-1, a novel noncoding RNA, and thymosin beta4 predict metastasis and survival in early-stage non-small cell lung cancer. Oncogene 22, 8031–8041 10.1038/sj.onc.1206928 12970751

[32] Rinn J.L., Kertesz M., Wang J.K., Squazzo S.L., Xu X. (2007) Functional demarcation of active and silent chromatin domains in human HOX loci by noncoding RNAs. Cell 129, 1311–1323 10.1016/j.cell.2007.05.022 17604720 PMC2084369

[33] Heo J.B. and Sung S. (2011) Vernalization-mediated epigenetic silencing by a long intronic noncoding RNA. Science 331, 76–79 10.1126/science.1197349 21127216

[34] Zhao J., Sun B.K., Erwin J.A., Song J.J. and Lee J.T. (2008) Polycomb proteins targeted by a short repeat RNA to the mouse X chromosome. Science 322, 750–756 10.1126/science.1163045 18974356 PMC2748911

[35] Yu W., Gius D., Onyango P., Muldoon-Jacobs K., Karp J. (2008) Epigenetic silencing of tumour suppressor gene p15 by its antisense RNA. Nature 451, 202–206 10.1038/nature06468 18185590 PMC2743558

[36] Poliseno L., Salmena L., Zhang J., Carver B., Haveman W.J. (2010) A coding-independent function of gene and pseudogene mRNAs regulates tumour biology. Nature 465, 1033–1038 10.1038/nature09144 20577206 PMC3206313

[37] Tseng R.C., Chang J.W., Hsien F.J., Chang Y.H., Hsiao C.F. (2005) Genomewide loss of heterozygosity and its clinical associations in non small cell lung cancer. Int. J. Cancer 117, 241–247 10.1002/ijc.21178 15900585

[38] (2016) A LincRNA risk variant promotes pancreatic tumorigenesis. Cancer Discov. 6, OF14 10.1158/2159-8290.CD-RW2016-101 27080335

[39] Shi X., Ma C., Zhu Q., Yuan D., Sun M. (2016) Upregulation of long intergenic noncoding RNA 00673 promotes tumor proliferation via LSD1 interaction and repression of NCALD in non-small-cell lung cancer. Oncotarget 7, 25558–25575 27027352 10.18632/oncotarget.8338 PMC5041926

[40] Ma C., Wu G., Zhu Q., Liu H., Yao Y. (2017) Long intergenic noncoding RNA 00673 promotes non-small-cell lung cancer metastasis by binding with EZH2 and causing epigenetic silencing of HOXA5. Oncotarget 8, 32696–32705 28423732 10.18632/oncotarget.16158 PMC5464820

