# Supplementary material for: LINC00673 rs11655237 C>T confers neuroblastoma susceptibility in Chinese population
Source: Biosci Rep. 2018 Feb 8;38(1):BSR20171667. doi: 10.1042/BSR20171667 (PMC5803493; doi:10.1042/BSR20171667)
Supplement: Supplementary file 1 [file bsr20171667_Supp1.pdf]

Supplemental Table 1. Frequency distribution of selected characteristics in neuroblastoma cases and cancer-free controls

Variables	Guangdong province					Henan province					Combined subjects				
	Cases (N=275)		Controls (N=531)		*P* [a]	Cases (N=118)		Controls (N=281)		*P* [a]	Cases (n=393)		Controls (n=812)		*P* [a]
	No.	%	No.	%		No.	%	No.	%		No.	%	No.	%	
Age range, month	0-132		0.07-156		0.229	0-131.1		0.1-144.0		0.484	0-132		0.07-156		0.437
Mean ± SD	31.50±25.43		29.73±24.86			46.24±29.98		44.97±33.23			35.92±27.68		35.01±28.94		
<12	70	25.45	145	27.31		9	7.63	32	11.39		79	20.10	177	21.80	
12-60	177	64.36	313	58.95		76	64.41	179	63.70		253	64.38	492	60.59	
>60	28	10.18	73	13.75		33	27.97	70	24.91		61	15.52	143	17.61	
Gender					0.510					0.196					0.836
Female	114	41.45	233	43.88		54	45.76	109	38.79		168	42.75	342	42.12	
Male	161	58.55	298	56.12		64	54.24	172	61.21		225	57.25	470	57.88	
Clinical stages															
I	54	19.64				15	12.71				69	17.56			
II	62	22.55				31	26.27				93	23.66			
III	49	17.82				19	16.10				68	17.30			
IV	94	34.18				49	41.53				143	36.39			
4s	8	2.91				3	2.54				11	2.80			
NA	8	2.91				1	0.85				9	2.30			
Sites of origin															
Adrenal gland	64	23.27				89	75.42				153	38.93			
Retroperitoneal region	87	31.64				/	/				87	22.14			
Mediastinum	90	32.73				19	16.10				109	27.74			
Other region	26	9.45				10	8.47				36	9.16			
NA	8	2.91				/	/				8	2.04			

SD, standard deviation; NA, not available.

[a] Two-sided χ^2 test for distributions between neuroblastoma cases and cancer-free controls.
